# Enhanced Performance of La_0.8_Sr_0.2_FeO_3-δ_-Gd_0.2_Ce_0.8_O_2-δ_ Cathode for Solid Oxide Fuel Cells by Surface Modification with BaCO_3_ Nanoparticles

**DOI:** 10.3390/mi13060884

**Published:** 2022-05-31

**Authors:** Halefom G. Desta, Yang Yang, Birkneh Sirak Teketel, Quan Yang, Kai Song, Shiyue Zhu, Dong Tian, Yonghong Chen, Tianyong Luo, Bin Lin

**Affiliations:** 1 School of Mechanical and Electrical Engineering, School of Materials and Energy, University of Electronic Science and Technology of China, Chengdu 611731, China; 21abeba21@gmail.com (H.G.D.); siraku642@gmail.com (B.S.T.); luotianyong@uestc.edu.cn (T.L.); 2 Anhui Province Key Laboratory of Low-Temperature Co-Fired Materials, Huainan Normal University, Huainan 232038, China; qyang3614@163.com (Q.Y.); gsjqsk@foxmail.com (K.S.); zhusy1@mail.ustc.edu.cn (S.Z.); tiandong111@163.com (D.T.); chenyh@hnnu.edu.cn (Y.C.); 3 School of Materials Science and Physics, China University of Mining and Technology, Xuzhou 221116, China; y-yang@cumt.edu.cn

**Keywords:** SOFCs, Fe-based perovskite oxide, impregnation, surface exchange kinetics, BaCO_3_

## Abstract

Recently, Fe-based perovskite oxides, such as Ln_1-x_Sr_x_FeO_3-δ_ (Ln = La, Pr, Nd, Sm, Eu) have been proposed as potential alternative electrode materials for solid oxide fuel cells (SOFCs), due to their good phase stability, electrocatalytic activity, and low cost. This work presents the catalytic effect of BaCO_3_ nanoparticles modified on a cobalt-free La_0.8_Sr_0.2_FeO_3-δ_-Gd_0.2_Ce_0.8_O_2-δ_ (LSF-GDC) composite cathode at an intermediate-temperature (IT)-SOFC. An electrochemical conductivity relaxation investigation (ECR) shows that the K_chem_ value of the modified LSF-GDC improves up to a factor of 17.47, demonstrating that the oxygen reduction process is effectively enhanced after surface impregnation by BaCO_3_. The area-specific resistance (ASR) of the LSF-GDC cathode, modified with 9.12 wt.% BaCO_3_, is 0.1 Ω.cm^2^ at 750 °C, which is about 2.2 times lower than that of the bare cathode (0.22 Ω.cm^2^). As a result, the anode-supported single cells, with the modified LSF-GDC cathode, deliver a high peak power density of 993 mW/cm^2^ at 750 °C, about 39.5% higher than that of the bare cell (712 mW/cm^2^). The single cells based on the modified cathode also displayed good performance stability for about 100 h at 700 °C. This study demonstrates the effectiveness of BaCO_3_ nanoparticles for improving the performance of IT-SOFC cathode materials.

## 1. Introduction

Energy consumption has been increasing globally, due to high population and demand growth. Fuel cells are energy conversion devices that provide enormous promise for delivering substantial environmental benefits. The solid oxide fuel cell (SOFC) is one of the most energy-efficient conversion devices: it can directly convert chemical energy stored in fuels into electricity with high conversion efficiency [1,2,3,4,5]. Unfortunately, the requisite high working temperatures hinder the commercialization of SOFC technology, by entailing various problems such as limiting the choice of materials, longer startup/shutdown times, and causing chemical and mechanical compatibility problems. Recently, extensive studies have been focused on reducing the working temperature of the SOFC to an intermediate operating temperature range (IT) [6,7]. Lowering the working temperature leads to an increase in the polarization resistance of the cathode materials. Therefore, extensive studies have been dedicated to developing cathode materials with high electrocatalysis at intermediate operating temperatures, to promote the commercialization of SOFC technology. Mixed ionic-electronic conductors (MIECs), especially cobalt-based perovskite oxides, have been widely studied as potential cathode materials of SOFCs, due to their high electronic/ionic conduction and catalytic activity [8,9,10,11]. However, cobalt-based materials suffer from poor structural stability, inadequate electrolyte compatibility, and relatively high price, which limit the practical application of SOFCs [12,13], necessitating the development of cobalt-free cathode material with high electrochemical performance in IT-SOFCs. Recently, Fe-based perovskite, such as Ln_1-x_Sr_x_FeO_3_ (Ln = La, Pr, Nd, Sm, Eu, and Gd) [14,15,16], and Ba_1-x_Sr_x_FeO_3_ [17,18], have shown promise as cathode materials for IT-SOFCs, owing to good catalytic activity for the ORR, high chemical stability, and comparatively low cost. Among ferrites-based materials, La_1-x_Sr_x_FeO_3-δ_ (LSF) has been extensively studied for its use as a potential cobalt-free cathode of SOFCs [19,20]. However, its performance is still inadequate compared to the requirements of cathode materials for IT-SOFCs. Consequently, several approaches have been proposed, to enhance the performance of LSF electrode materials for IT-SOFCs, such as developing composite cathodes, doping other metal ions, and surface modification [21,22,23]. 

Surface modification of La_0.8_Sr_0.2_FeO_3-δ_ (LSF) cathode materials via impregnation of nanoparticles are an effective strategy for enhancing the electrochemical performance of SOFCs. For example, Qiuyun Lin, et al., [24] reported a peak power density (MPD) of 726 mW/cm^2^ at 750 °C on a La_0.6_Sr_0.4_CoO_3-δ_ (LCF)-impregnated LSF-GDC cathode, much higher than that of the bare cathode (298 mW/cm^2^). Tao Hong, et al., [25], also reported an MPD of 0.53 mW/cm^2^ at 800 °C, for a BaCO_3-_infiltrated LSF cathode, higher than that of a cell with a bare LSF cathode (0.3 mW/cm^2^) and the polarization resistance was reduced to 0.84 Ω.cm^2^ at 700 °C. However, to the best of our knowledge, the surface modification of an LSF-GDC composite cathode via the impregnation of BaCO_3_ has not been reported to date.

This study reports the pronounced performance of LSF-GDC cathodes of IT-SOFCs via impregnation by BaCO_3_ nanoparticles. The most common BaCO_3_ nanoparticle was chosen as the impregnating catalyst, due to its being widely accessible at a very low cost [26]. The ECR was investigated to examine the catalytic effect of BaCO_3_ on the ORR kinetics of the LSF-GDC cathode. The ASR of bare and BaCO_3_ modified cathodes was studied, using the symmetrical cell of LSF-GDC|GDC|YSZ|GDC|LSF-GDC, and the performance of the cathodes was evaluated using an anode-supported single cell. The surface-modified LSF-GDC cathode with BaCO_3_ nanoparticle is expected to achieve high electrochemical performance.

## 2. Experimental Section

### 2.1. Materials Preparation

LSF and GDC powders were prepared via a modified Pechini method [27]. Stoichiometric amounts of La(NO_3_)_3_, Sr(NO_3_)_2,_ and Fe(NO_3_)_3_·9H_2_O were dissolved in distilled water. Subsequently, citric acid was added, and the molar ratio of total metal ion to citric acid was 1:1.5. Ammonia solution was added to adjust the pH value of the solution to about 7–8, and the obtained precursor was heated in an electric oven until self-combustion was complete. The GDC powders were also prepared through the above procedure, where Gd (NO_3_)_3_ and Ce(NO_3_)_3_·6H_2_O were used as precursors of metal nitrates. The porous ash products were calcined at 700 and 1000 °C for 3 h, to form GDC and LSF powders with fluorite structure and perovskite structure, respectively. Finally, the as-prepared GDC and LSF powders (40:60 by weight ratio) were mixed using a ball mill to form LSF-GDC composite powders.

### 2.2. Cell Fabrication and Testing

Symmetrical cells of LSF- GDC|GDC|YSZ|GDC|LSF-GDC were prepared and employed for electrochemical impedance measurement. The commercially available YSZ powders were dry pressed under 250 MPa to form pellets, and then sintered at 1450 °C for 10 h. The LSF-GDC and GDC powders were mixed with 10 and 5 wt.% ethyl cellulose, respectively, in terpineol, to prepare the compounding slurries. The as-prepared GDC slurries were symmetrically spin-coated onto the YSZ pellet, and sintered at 1300 °C for 3 h. The LSF-GDC slurries were coated on the surface of the GDC layer and finally sintered at 1000 °C for 3 h, to obtain the symmetrical cell where the area of the applied electrode was about 0.567 cm^2^. Electrochemical impedance spectroscopy (EIS) test was carried out, using an IM6 electrochemical workstation (ZAHNER, Germany) from 0.01 Hz to 1 MHz, over a temperature range of 650–750 °C with an AC amplitude of 5 mV. 

Anode-supported single cells of NiO-YSZ|YSZ|GDC|LSF-GDC were fabricated and applied for electrochemical performance measurement. The well-mixed of NiO, YSZ powder, and starch, with a 60:40:20 weight ratio respectively, were pressed to form green pellets, and sintered at 1000 °C for 3 h. The YSZ slurries were prepared using a similar method as for the buffer slurries. The electrolyte slurry was subsequently coated three times on the NiO-YSZ anode support, and each coating was heated at 450 °C for 30 min, followed by sintering at 1400 °C for 10 h. The GDC buffer layer slurries were then coated onto the YSZ surface, and sintered at 1300 °C for 3 h. Finally, the LSF-GDC slurries were coated onto the buffer layer’s surface and sintered at 1000 °C for 3 h. The fuel cells’ performance was measured from 650 to 750 °C, with humidified H_2_ as fuel and ambient air as oxidant.

An appropriate amount of barium acetate (Ba (OAc)_2_) was dissolved in a mixed solvent (distilled water 1:1 ethanol) to prepare 0.3 mol. L^−1^ Ba (OAc)_2_ as a precursor solution for the impregnation process. The as-prepared Ba (OAc)_2_ precursor solution was dropped onto the surface of the LSF-GDC using a pipette, and dried in a vacuum chamber, then heated at 800 °C for 2 h in air. The loading of the BaCO_3_ was estimated by measuring the weight change of the cathode after and before each impregnation process. 

### 2.3. Characterizations

The thermal decomposition temperatures of the Ba(OAc)_2_ were characterized using a thermo-gravimetric analysis instrument. The measurement was carried out in the air atmospheres from room temperature to 800 °C, with a heating rate of 10 min^−1^. The crystal structures of the synthesized sample were analyzed by X-ray diffraction (XRD, DX-2800), using Cu-Ka radiation with a 2θ range from 20° to 80°. The microstructure and elemental mapping analysis of the sample were performed using a Gemini 300, field emission scanning electron microscope (FESEM), and energy-dispersive X-ray spectroscopy (EDS, Oxford), respectively. For the electrical conductivity relaxation study, the LSF-GDC powders were pressed at 300 MPa, followed by sintering at 1400 °C for 5 h, to obtain dense LSF-GDC samples with approximate dimensions of 1.2 mm × 4.8 mm × 10 mm. The electrical conductivity relaxation test was conducted using a dc four-probe technique. While the oxygen partial pressure shifted from P_O2_ = 0.2 bar (P_O2_ = 0.2 and P_N2_ = 0.0) to P_O2_ = 0.1 bar (P_O2_ = 0.1 and P_N2_ = 0.1), the change in electrical conductivity as function time was recorded. The surface exchange coefficient (k_chem_) was estimated by fitting electrical conductivity relaxation curves.

## 3. Results and Discussion

### 3.1. Thermal Analysis and Chemical Compatibility

**Figure 1:**
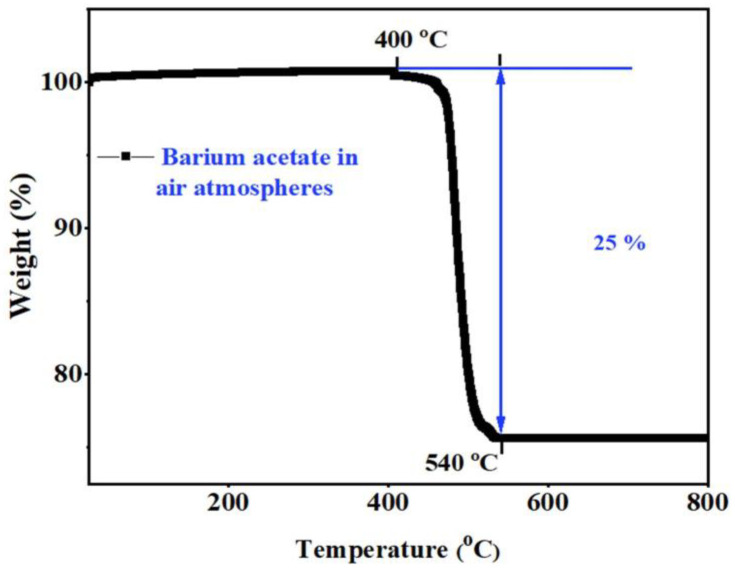
Thermogravimetric curves of Ba(OAc)_2_.

Figure 1 shows the thermal decomposition of Ba(OAc)_2_ in the temperature range of 25–800 °C in air. The weight loss started from 400 °C up to 534 °C; approximately 25% weight loss was recorded. The weight loss may have corresponded to the removal of moisture and carbon dioxide. The weight loss percentage was close to the theoretical value of the decomposition reaction of Ba(Ac)_2_ = BaCO_3_ + CO_2_ + H_2_O [28,29]. Furthermore, no extra weight loss was observed between 534 and 800 °C, and this may have been associated with the formation of BaCO_3_, which was stable in the operating temperature range. 

**Figure 2:**
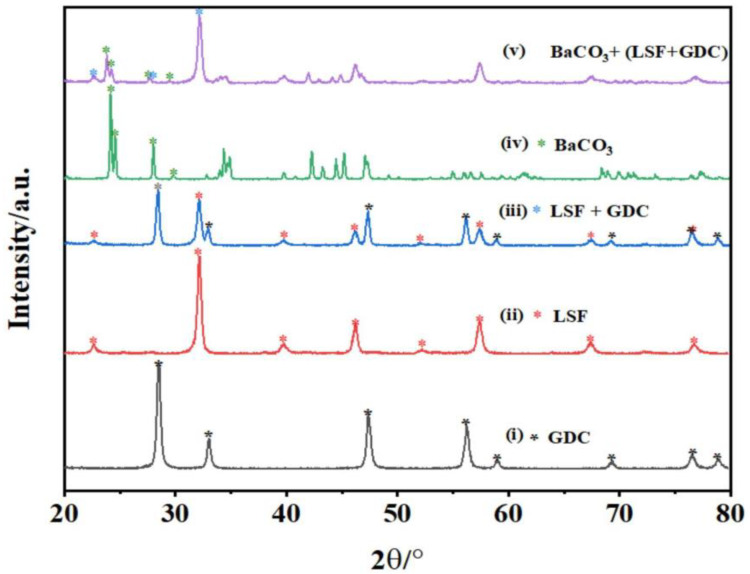
XRD patterns of the LSF and GDC powders, and the composite of LSF + GDC and BaCO_3_ + (LSF + GDC).

The XRD patterns of the powders and the chemical compatibility of the mixed powders of the LSF + GDC and BaCO_3_ + (LSF + GDC) are presented in Figure 2. After calcination of the GDC powder at 700 °C in air, the XRD patterns exhibited a cubic fluorite phase (PDF# 50-0201), and the refined lattice parameters were a = b = c 5.411 Å, with the space group Fm-3 m, as shown in Figure 2i. Figure 2ii displays the XRD patterns of LSF powders calcined at 1000 °C. As is shown, all peaks can be indexed in an orthorhombic perovskite structure (PDF # 35-1480), and the refined lattice parameters were a = 5.532 Å, b = 5.553 Å, c = 7.835 Å, with the space group Pbn (62). The chemical compatibility between LSF and GDC was analyzed by mixing LSF and GDC powders at a weight ratio of 1:1 and heating at 1000 °C for 5 h in the air. The diffraction peaks of the LSF + GDC composite powder show that no obvious solid-state reaction was observed between LSF and GDC, as shown in Figure 2iii. Barium acetate (Ba(OAc)_2_ was thermally heated at 800 °C for 2 h, and this led to the formation of orthorhombic structured BaCO_3_ with a space group of Pmcn (62) as shown in Figure 2iv. Next, (LSF + GDC) and Ba (OAc)_2_ powders were mixed (50:50 wt.% in weight ratio) and co-heated at 800 °C for 5 h in air, to analyze the chemical compatibility between the composite cathode and BaCO_3_ powders. As shown in Figure 2v, the peaks belong to either BaCO_3_ or (LSF + GDC), and this could indicate that Ba (OAc)_2_ thermally decomposes into BaCO_3_ in the presence of (LSF + GDC), without any solid-state reaction being observed between the two-phase. These results also indicate that a BaCO_3_-impregnated (LSF + GDC) cathode, can be successfully prepared by the impregnation of Ba (OAc)_2_, and could be stable during the conduction testing.

### 3.2. Microstructure Analysis

**Figure 3:**
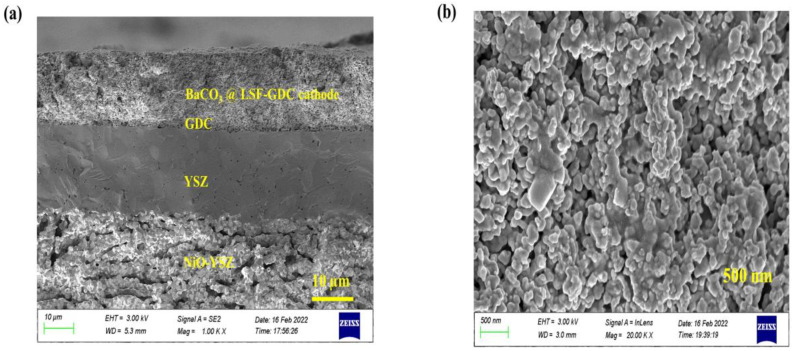
SEM images of (**a**) NiO-YSZ|YSZ|GDC|LSF-GDC and (**b**) BaCO_3_ nanoparticles modified LSF-GDC cathode.

**Figure 4:**
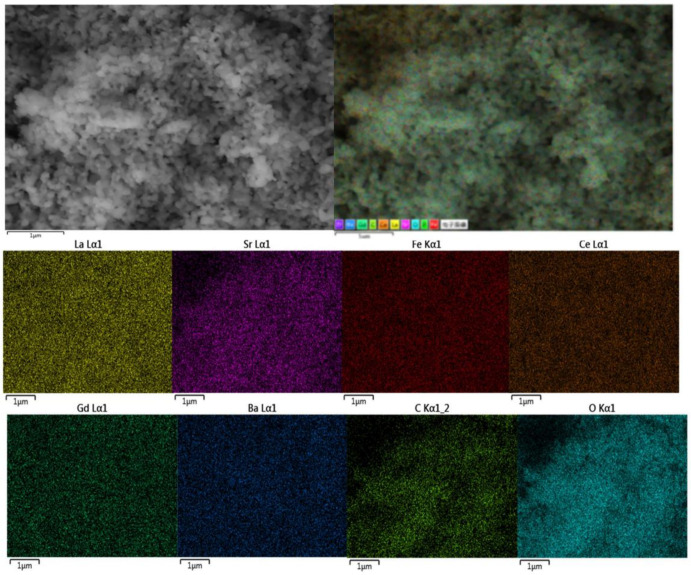
EDS elemental mappings of the modified LSF-GDC.

Figure 3a shows a typical SEM image of anode-support single cells with thicknesses of electrolyte (YSZ) ~18 μm and buffer layer (GDC) ~2 µm. The YSZ electrolyte layer has a suitable density and is well-adhered to both the GDC layer and NiO-YSZ anode supports, which effectively block the gas component diffusion to the opposite electrode. The microstructure of the targeted cathodes attaches well to the buffer layer (GDC) surface. The BaCO_3_ nanoparticles are distributed on the surface of the cathode; which can be seen in Figure 3b. The porosity of the pre-existing cathode remains unchanged. The element mapping image of an impregnated LSF-GDC cathode was obtained using EDS, as shown in Figure 4. The EDS results show that the elements including La, Sr, Fe, Ce, Gd, Ba, C, and O are uniformly distributed in the impregnated cathode. The presence of Ba and C, within the surface of the cathode, suggests that BaCO_3_ was successfully impregnated on the surface of the LSF-GDC composite cathode. 

### 3.3. Electrochemical Activity

**Figure 5:**
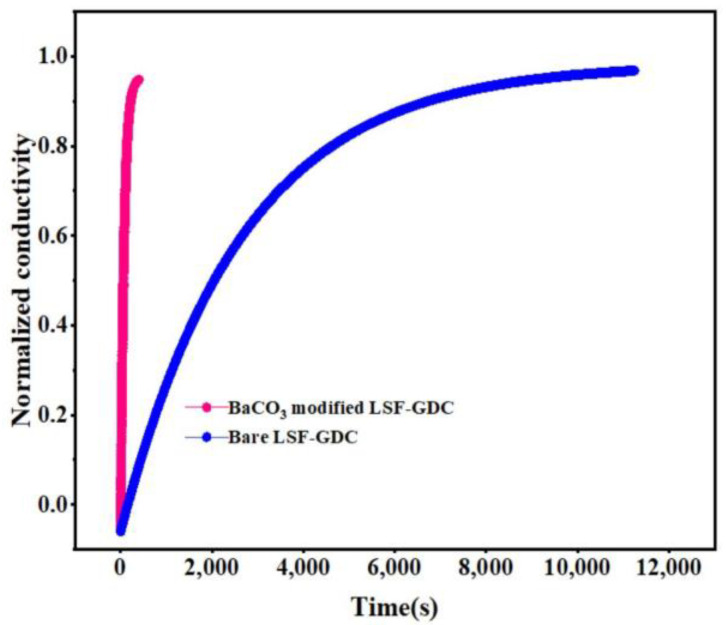
The electrical conductivity relaxation responses curves of bare LSF-GDC and BaCO_3_-modified LSF-GDC bars at 750 °C.

Abrupt oxygen partial pressure causes oxygen incorporation within the cathode materials and in turn, changes the cathode materials’ conductivity. The oxygen surface exchange capacity of cathode materials suggests the adsorption and dissociation rate of the oxygen, which affects the reaction kinetics of the materials. Figure 5 shows the normalized conductivity response curves of the bare LSF-GDC and BaCO_3_-modified LSF-GDC samples after abruptly switching the oxygen partial pressure (PO_2_) from 0.21 to 0.1 atm at 750 °C. The relaxation time, to reach re-equilibrium, for 0.65 mg/cm^2^ BaCO_3_-modified LSF-GDC after P_O2_ abruptly switching, was ~400 s, which is 27.5 times faster than the bare LSF-GDC (11,000 s). The faster re-equilibrium time may have been attributable to the enhancement of the surface oxygen exchange process since the bulk diffusion rate should have been the same for the two samples (bare and surface-modified); as a result, the oxygen surface exchange coefficient (K_chem_) value of the modified LSF-GDC was 2.194 × 10^−4^ cms^−1^. However, the K_chem_ value of the bare sample was 1.256 × 10^−5^ cms^−1^. The K_chem_ value of the modified LSF-GDC improved up to a factor of 17.47, demonstrating that the oxygen reduction process was effectively enhanced after impregnation by the BaCO_3_ nanoparticles, and this concurred with the EIS values. The reduced re-equilibrium time and enhanced oxygen surface exchange coefficient may have been associated with the synergistic oxygen reduction reaction catalytic activity of BaCO_3_ nanoparticles. The K_chem_ value modified LSF-GDC was comparable with previously reported LSF/LSCF materials modified by SrCO_3_, CaO, MgO, and BaCO_3,_ as shown in Table 1. However, a further theoretical study is required, to reveal the enhancement mechanism. 

**Figure 6:**
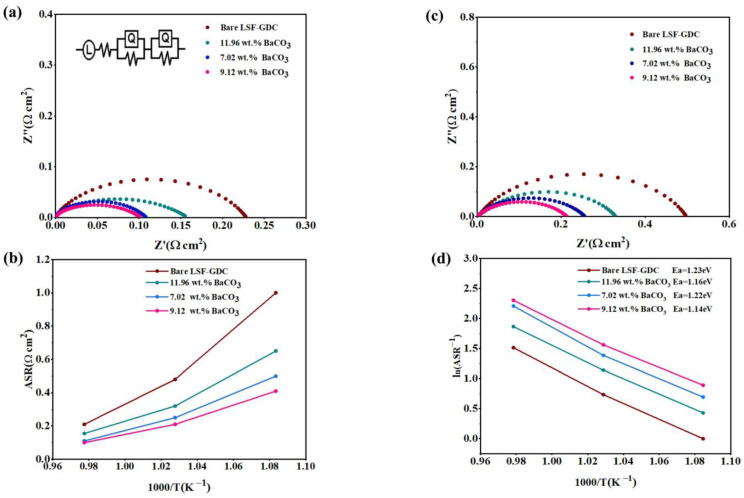
Comparison of EIS spectra for different loadings of BaCO_3_ on LSF-GDC at (**a**) 750 °C, (**b**) 700 °C, (**c**) ASR, as a function of temperature, and (**d**) corresponding Arrhenius plots of ASR.

The EIS spectra of the symmetric cell LSC-GDC|YSZ|LSF-GDC with different loadings of BaCO_3_ are shown in Figure 6. For simple analysis, the ohmic impedances that come from the electrolyte and silver wires were subtracted. The experimental data were fitted by the electrical equivalent circuit model of L–Ro-(R_H_Q_H_)-(R_L_Q_L_), where, L, Ro, R_H_Q_H_, and R_L_Q_L_, represent inductance, ohmic impedance, a high frequency, and low-frequency arc, respectively. The area-specific resistance (ASR) of the cathode was estimated from the difference between the total impedance of the cell and the ohmic impedance. The ASR values at 750 °C were determined as 0.31, 0.15, 0.11, and 0.1 Ω.cm^2^ at 750 °C for bare LSF-GDC, and 11.96, 7.02, and 9.12 wt.% BaCO_3_, respectively, as presented in Figure 6a. The lowest ASR values were attained with optimum loading of 9.12 wt.% BaCO_3_ at different temperatures. The ASR values decreased from 0.48 to 0.21 Ω.cm^2^ at 700 °C, and 0.31 to 0.1 at 750 °C, when 9.12 wt.% BaCO_3_ nanoparticles were loaded. However, when the loading further increased to 11.96 wt.% BaCO_3_, the ASR slightly increased to 0.15 Ω.cm^2^: this may have been associated with the blockage of the active pore of the cathode with inert BaCO_3_ nanoparticles, which led to a delay in the diffusion of oxygen to the active area. The enhancement performance of the LSF-GDC cathode may have been associated with the accelerated oxygen surface exchange process after the impregnation of the optimum BaCO_3_. Figure 6d demonstrates the Arrhenius plot for the ASR values of the different BaCO_3_ nanoparticles’ loading and the corresponding activation energy values. The activation energies (E_a_), calculated from the Arrhenius plots, were 1.228, 1.163, 1.226, and 1.143 eV for bare LSF-GDC, and 11.96, 7.02, and 9.12 wt.% BaCO_3_, respectively. The lowest activation energy was achieved for 9.12 wt.% BaCO_3_, indicating that the oxygen reduction process was effectively enhanced with the optimum loading of BaCO_3_ nanoparticles.

### 3.4. Cell Performance

**Figure 7:**
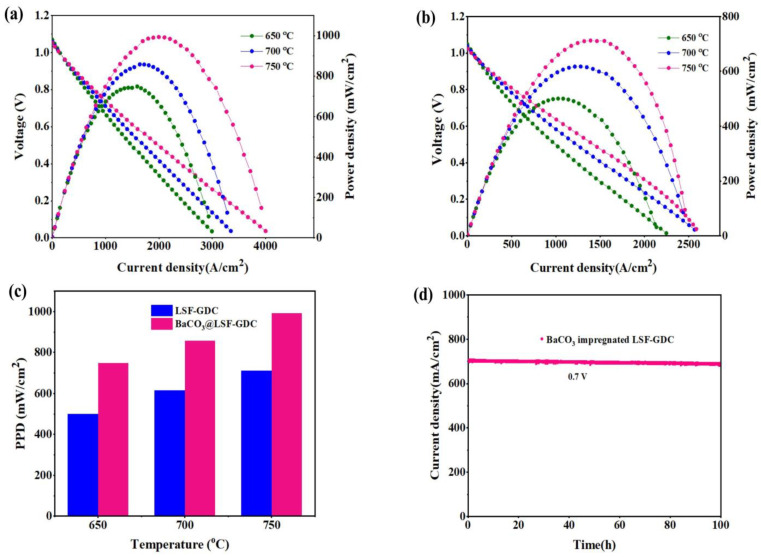
Performance of anode-supported cell NiO-YSZ|YSZ|GDC|LSF-GDC with (**a**) 8.21 wt.% BaCO_3_-modified LSF-GDC cathode, (**b**) bare LSF-GDC cathode, (**c**) comparative peak power densities (PPD), and (**d**) stability test at 700 °C, under fixed voltage (0.7 V).

Figure 7 displays the performance of a single cell measured between 650 and 750 °C. The open-circuit voltage (OCV) varied from ~1.031 to ~1.047 V, close to the theoretical value, suggesting that the YSZ electrolyte was completely dense. The peak power densities (PPD), with an 8.21 wt.% BaCO_3_-modified LSF-GDC cathode, were 993, 858, and 748 mW/cm^2^ at 750, 700, and 650 °C, respectively, as shown in Figure 7a. The peak power densities (PPD) of a single cell with a bare LSF-GDC cathode were 712, 617, and 501 mW/cm^2^ at 750, 700, and 650 °C, respectively, as displayed in Figure 7b. Comparatively, the peak power densities (PPD) of the modified cathode were much higher than that of the bare cathode, as shown in Figure 7c. For example, the peak power densities at 750 °C were 712 and 993 mW/cm^2^ for the bare and for the modified LSF-GDC, respectively, enhanced by 39.5% via the impregnation of BaCO_3_ nanoparticles. This enhancement was significantly higher compared to the previous report on anode-supported cells based on LSF/LSF-GDC cathodes [22,25,33]. Figure 7d displays the stability of NiO-YSZ-anode-supported SOFC with modified LSF-GDC cathode under a fixed voltage of 0.7 V. It was found that the measured current density of the single-cell was kept stable at ~700 mA/cm^2^, indicating excellent performance stability for about 100 h at 700 °C.

Recently, the La_0.6_Sr_0.4_Co_0.2_Fe_0.8_O_3-δ_-Gd_0.2_Ce_0.8_O_2-δ_ composite cathode has been widely studied as the state-of-the-art composite cathode material of SOFC at reduced temperature, due to its high electrical and satisfactory ionic conductivities properties.

Comparatively speaking, the peak power densities (PPD) of a single ell with BaCO_3_ modified LSF-GDC are comparable with the state-of-the-art LSCF-GDC composite cathode at 750 and 700 °C, as shown in Table 2. At 650 °C, the performance observed in this study was superior to that of the LSCF-GDC composite cathode. The results indicate the vital role of the impregnation of BaCO_3_ nanoparticles in enhancing the performance of SOFC cathode materials at a reduced temperature.

Recent studies have shown that surface modification of cathode materials with alkali earth metal compounds greatly enhances electrochemical activity [30,31,32]. The most common BaCO_3_ nanoparticle has demonstrated outstanding catalytic activity toward oxygen reduction reaction for IT-SOFCs [26]. LSF-GDC modified by 0.65 mg/cm^2^ BaCO_3_ could enhance the oxygen exchange coefficient from 1.256 × 10^−5^ to 2.194 × 10^−4^ cms^−1^ at 750 °C. An electrochemical conductivity relaxation investigation (ECR) showed that the K_chem_ value of the impregnated LSF-GDC was improved up to a factor of 17.47, demonstrating that the oxygen reduction process is effectively enhanced after impregnation by BaCO_3_. The pronounced performance of the LSF-GDC cathode could be attributed to the enhanced kinetics. A theoretic study is required to reveal the enhancement mechanism. Furthermore, our studies can be extended to low-temperature solid-oxide fuel cells.

## 4. Conclusions

In summary, the impregnation of barium acetate (Ba(OAc)_2_) process results in BaCO_3_ nanoparticles on the LSF-GDC surface, with no solid-state reaction being observed between the two phases. The modified LSF-GDC sample with 0.65 mg/cm^2^ BaCO_3_ enhanced the oxygen surface exchange process kinetics values (K_chem_) from 1.256 × 10^−5^ to 2.194 × 10^−4^ cms^−1^ at 750 °C. As compared to the bare LSF-GDC cathode, the surface-modified LSF-GDC cathode significantly enhanced the catalytic activity, as proved by lower ASRs and higher power densities. The improvement in electrochemical performance may be attributable to the accelerated oxygen surface exchange process through BaCO_3_ impregnation. Furthermore, the single cells based on the modified cathode displayed excellent performance stability under a fixed voltage of 0.7 V for more than 100 h at 700 °C. The performance of the single-cell was comparable to the most extensively studied LSCF-GDC composite cathode of IT-SOFC. The electrochemical results reported here indicate that such a simple and cost-effective strategy is a potential approach to improving the performance of IT-SOFC cathodes, although further theoretic study is still required, to reveal the detailed mechanism for the electrochemical activity of BaCO_3_ nanoparticles for the oxygen reduction reaction.

## Figures and Tables

**Table 1:** Comparison of the K_chem_ (cm s^−1^) of LSF/LSCF modified with alkali earth metal compounds.

**Bare**	K_chem_	Modified with	K_chem_	T(°C)	Ref.
La_0.8_Sr_0.2_FeO_3-δ_-Gd_0.2_Ce_0.8_O_2-δ_	1.256 × 10^−5^	0.65 mg/cm^2^ BaCO_3_	2.194 × 10^−4^	750	This work
La_0.8_Sr_0.2_FeO_3-δ_	1 × 10^−5^	0.95 mg/cm^2^ BaCO_3_	9.9 × 10^−5^	700	[25]
La_0.6_Sr_0.4_Co_0.2_Fe_0.8_O_3-δ_	1.8 × 10^−5^	0.85 mg/cm^2^ BaCO_3_	1.5 × 10^−4^	700	[26]
La_0.6_Sr_0.4_Co_0.2_Fe_0.8_O_3-δ_	4.0 × 10^−5^	0.055 mg/cm^2^ MgO	9.49 × 10^−5^	750	[30]
La_0.6_Sr_0.4_Co_0.2_Fe_0.8_O_3-δ_	1.8 × 10^−5^	0.07 mg/cm^2^ CaO	2.81 × 10^−4^	700	[31]
La_0.6_Sr_0.4_Co_0.2_Fe_0.8_O_3-δ_	2.2 × 10^−5^	SrCO_3_	2.4 × 10^−3^	700	[32]

**Table 2:** Comparison of the peak power densities (mW/cm^2^) of BaCO_3_-modified LSF-GDC composite cathode with state-of-the-art LSCFGDC composite cathode.

Anode	Electrolyte	Buffer Layer	Cathode	PPD (650 °C mW/cm^2^)	PPD (700 °C mW/cm^2^)	PPD (750 °C mW/cm^2^)	Ref.
NiO-YSZ	YSZ	GDC	BaCO_3_@LSF-GDC	748	858	993	This work
NiO-YSZ	YSZ	GDC	LSCF-GDC	410	680	1030	[34]
NiO-YSZ	YSZ	GDC	LSCF-GDC	380	650	1020	[35]
NiO-YSZ	YSZ	GDC	LSCF-GDC	-	540	990	[36]
NiO-YSZ	YSZ	GDC	LSCF-GDC	580	840	1080	[37]
NiO-YSZ	YSZ	GDC	LSCF-GDC	562			[38]

## Data Availability

Not applicable.
